# Network-directed cis-mediator analysis of normal prostate tissue expression profiles reveals downstream regulatory associations of prostate cancer susceptibility loci

**DOI:** 10.18632/oncotarget.20717

**Published:** 2017-09-08

**Authors:** Nicholas B. Larson, Shannon K. McDonnell, Zach Fogarty, Melissa C. Larson, John Cheville, Shaun Riska, Saurabh Baheti, Alexandra M. Weber, Asha A. Nair, Liang Wang, Daniel O’Brien, Jaime Davila, Daniel J. Schaid, Stephen N. Thibodeau

**Affiliations:** ^1^ Division of Biomedical Statistics and Informatics, Department of Health Sciences Research, Mayo Clinic, Rochester, MN, USA; ^2^ Division of Anatomic Pathology, Department of Laboratory Medicine and Pathology, Mayo Clinic, Rochester, MN, USA; ^3^ Department of Computational Medicine and Bioinformatics, University of Michigan, Ann Arbor, MI, USA; ^4^ Department of Pathology, Medical College of Wisconsin, Milwaukee, WI, USA; ^5^ Division of Laboratory Genetics, Department of Laboratory Medicine and Pathology, Mayo Clinic, Rochester, MN, USA

**Keywords:** prostate cancer, genetic risk, expression quantitative trait loci, mediation

## Abstract

Large-scale genome-wide association studies have identified multiple single-nucleotide polymorphisms associated with risk of prostate cancer. Many of these genetic variants are presumed to be regulatory in nature; however, follow-up expression quantitative trait loci (eQTL) association studies have to-date been restricted largely to *cis*-acting associations due to study limitations. While *trans*-eQTL scans suffer from high testing dimensionality, recent evidence indicates most *trans*-eQTL associations are mediated by *cis*-regulated genes, such as transcription factors. Leveraging a data-driven gene co-expression network, we conducted a comprehensive *cis*-mediator analysis using RNA-Seq data from 471 normal prostate tissue samples to identify downstream regulatory associations of previously identified prostate cancer risk variants. We discovered multiple *trans*-eQTL associations that were significantly mediated by *cis*-regulated transcripts, four of which involved risk locus 17q12, proximal transcription factor *HNF1B*, and target *trans*-genes with known HNF response elements (*MIA2*, *SRC*, *SEMA6A*, *KIF12*). We additionally identified evidence of *cis*-acting down-regulation of *MSMB* via rs10993994 corresponding to reduced co-expression of *NDRG1*. The majority of these *cis*-mediator relationships demonstrated *trans*-eQTL replicability in 87 prostate tissue samples from the Gene-Tissue Expression Project. These findings provide further biological context to known risk loci and outline new hypotheses for investigation into the etiology of prostate cancer.

## INTRODUCTION

Prostate cancer (PRCA) is one of the most heritable cancers, with latest estimates of the genetic contribution to total risk near 58% [1]. To date, a total of 202 PRCA risk-associated loci have been reported by genome-wide association studies [2–16], which collectively explain approximately one third of the total familial risk. The majority of these variants does not occur within genic regions and are presumed to be regulatory in nature. Multiple expression quantitative trait loci (eQTL) studies have investigated associations between PRCA susceptibility loci and transcript expression levels of nearby genes [17, 18]. These studies have identified a large number of dysregulated genes that may be relevant to the development and progression of PRCA.

Due to the high testing-dimensionality presented by evaluating transcriptome-wide associations, most eQTL studies of trait-associated genetic variation focus on *cis*-acting regulation (*cis*-eQTLs). Thus, tested associations are limited to genes near the variants of interest. However, a growing number of studies have identified *trans*-eQTLs are also likely to play a major role in disease etiology [19–21]. As transcriptional regulation is highly determined by cell-type, large tissue-specific datasets and sophisticated methods are necessary to discover *trans*-associations of trait-associated loci. For example, Yao et al. [22, 23] have leveraged a large whole-blood eQTL dataset from the Framingham Heart Study to investigate the role of both *cis*- and *trans*-eQTLs among SNPs associated with cardiometabolic traits relevant to cardiovascular disease. In PRCA, Chen et al. [24] applied a Bayesian clustering approach toward investigating the role of *trans*-associations of reported risk loci in a relatively small set of tumor-adjacent stromal tissue samples. Other approaches, including adaptive false discovery rate estimation [25] and cross-phenotype meta-analysis [26], have focused on *trans*-eQTL “hotspots”, whereby genetic loci are associated in *trans* with expression levels of multiple transcripts.

Multiple studies have indicated *trans* associations are likely mediated by the products of *cis*-regulated transcripts [22, 27], such as transcription factors and signaling cascade proteins. A plausible strategy to improve discovery of PRCA risk SNP *trans*-eQTL associations under such a model is leveraging patterns of gene co-expression with *cis*-regulated genes. Gene co-expression analysis is a powerful data-driven approach for uncovering relevant regulatory networks in high-dimensional expression data. Recent improvements in the construction of sparse undirected graphs using regularized Gaussian graphical models have enabled sparse network inference on large gene expression datasets [28]. In this study, we systematically investigate potential downstream *trans*-acting dysregulation of protein-coding gene expression by PRCA risk loci using *cis*-mediator analysis on a large prostate tissue eQTL dataset. We first substantially reduce the search space of *trans*-eQTL associations by constructing an undirected co-expression network of genes exhibiting at least modest eQTL associations with PRCA risk loci. We then identify *cis*-eQTL genes as potential mediators of *trans*-eQTL associations. We then apply a network-driven strategy to determine if neighboring genes in the expression graph exhibit *trans*-eQTL associations that are mediated by *cis*-regulatory effects using causal inference analyses. Finally, we interpret putative regulatory targets of dysregulated *cis*-eQTL genes in the context of PRCA susceptibility.

## RESULTS

**Figure 1:**
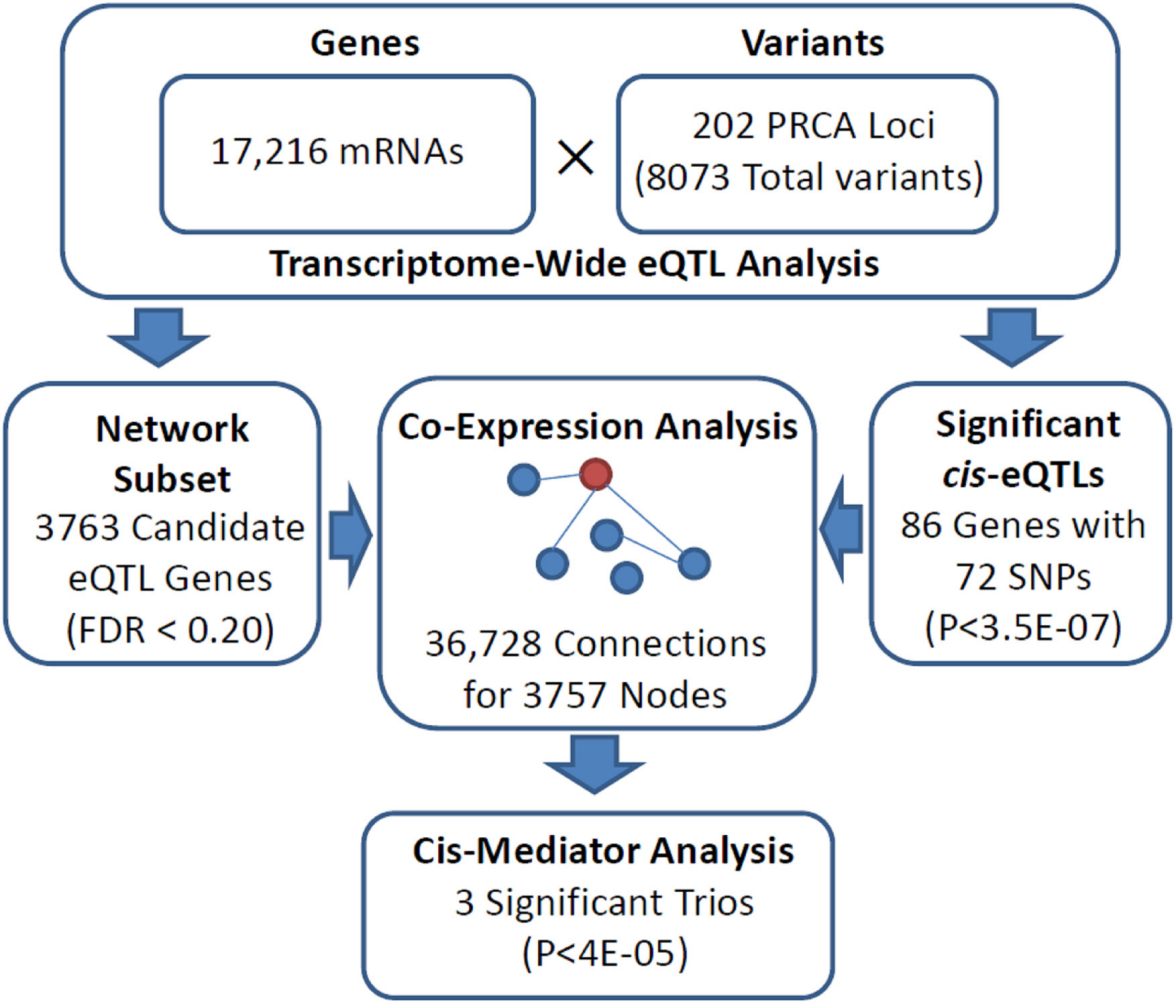
Flowchart indicating analytical stages in identifying significant cis-mediator relationships between PRCA susceptibility loci, proximal *cis*-genes, and distal *trans*-genes

A total of 3763 expressed transcripts met our transcriptome-wide eQTL screening criteria for inclusion in the co-expression network inference (FDR < 0.2). The estimated undirected graph for this gene subset consisted of 36,728 connections involving 3757 unique transcripts. Of the 3130 candidate *cis*-eQTL target genes, we identified 86 significant *cis*-genes associated with 72 unique PRCA risk loci variants (Supplementary Table 1). A total of 1168 neighbor nodes of the significant *cis*-genes in the expression network met our definition of *trans* with the corresponding *cis*-eQTL variant, defining *cis*-mediator trios eligible for causal inference. Of these, three *cis*-mediator trios resulted in mediation p-values below the Bonferroni-adjusted significance threshold of 0.05/1168 ≈ 4.3E-05: rs11263762→*HNF1B*→*SRC*, rs11263762→*HNF1B*→*MIA2*, and rs10993994→*MSMB*→*NDRG1*. A flowchart of these analyses is represented in Figure 1, while complete results for seven trios that exhibited at least suggestive associations (mediation P < 1E-03) are presented in Table 1. Two additional genes corresponded to suggestive *cis*-mediator relationships with rs11263762 and *HNF1B*: *KIF12* (mediation P = 1.2E-04) and *SEMA6A* (mediation P = 3.0E-04). As *HNF1B* encodes the transcription factor HNF-1B, these results highlight multiple putative targets of *trans*-acting dysregulation via PRCA risk SNP rs11263762.

**Table 1:** Significant and suggestive (mediation P < 1E-05) *cis*-mediator analysis results from the PRCA susceptibility gene expression network

eQTL Variant			*Cis*-Gene			*Trans*-Gene			Mediation Analysis		
rsID	Chr:Pos (hg19)	Alleles^a^	Gene	*β*_*C*_	*P*_*C*_	Gene	*Β*_*T*_	*P*_*T*_	*P*	$\beta_{T}^{adj}$	*M*
rs11263762	17: 36101926	G/A	*HNF1B*	0.15	4.4E-12	*SRC*	0.04	3.5E-07	3.5E-07	0.02	0.63
rs11263762	17: 36101926	G/A	*HNF1B*	0.15	4.4E-12	*MIA2*	0.21	1.2E-06	1.2E-06	0.10	0.55
rs10993994	10: 51549496	C/T	*MSMB*	-0.32	7.4E-38	*NDRG1*	-0.10	1.0E-05	1.0E-05	-0.02	0.83
rs11191385	10: 104513049	G/T	*AS3MT*	-0.22	1.0E-32	*TMEM121*	-0.08	3.5E-05	1.2E-04	-0.03	0.56
rs11263762	17: 36101926	G/A	*HNF1B*	0.15	4.4E-12	*KIF12*	0.13	1.2E-04	1.2E-04	0.04	0.66
rs11263762	17: 36101926	G/A	*HNF1B*	0.15	4.4E-12	*SEMA6A*	0.14	2.4E-10	3.0E-04	0.07	0.50
rs6958572	7:97789351	G/A	*TECPR1*	0.08	3.8E-13	*UBA5*	0.02	4.1E-04	9.9E-04	0.01	0.58

Reference and alternate alleles were defined via frequency in the dataset.

^a^Major/Minor as defined by observed minor allele frequency among 471 prostate tissue samples.

Quantification of eQTL effect mediation by *M* for significant *cis*-mediator relationships indicated incomplete attenuation of the *trans*-eQTL by the mediating *cis*-genes, ranging from 0.50 to 0.83 (Supplementary Figure 1). However, this is not altogether surprising, as such partial mediation is expected in the presence of measurement error and incorrect selection of the underlying causal regulatory variant due to LD [27]. To further evaluate the robustness of these associations, we conducted permutation testing as similarly conducted in Franzen et al. [29]. Briefly, for each *cis*-mediator trio, *trans*-gene expression values were randomly permuted across samples within a given genotype group defined by levels of *L*, holding all other measurements fixed. The mediation analysis p-values from the permuted datasets were compared to the original results and permutation p-values were computed as the proportion of permuted data p-values as or more extreme than the p-value derived from the true data. Under 100,000 permutations and the same significance threshold defined above, all reported associations in Table 1 corresponded to the minimum permutation p-value (1.0E-05) and declared as significant aside from rs11263762→*HNF1B*→*SEMA6A* (P = 1.5E-04).

**Figure 2:**
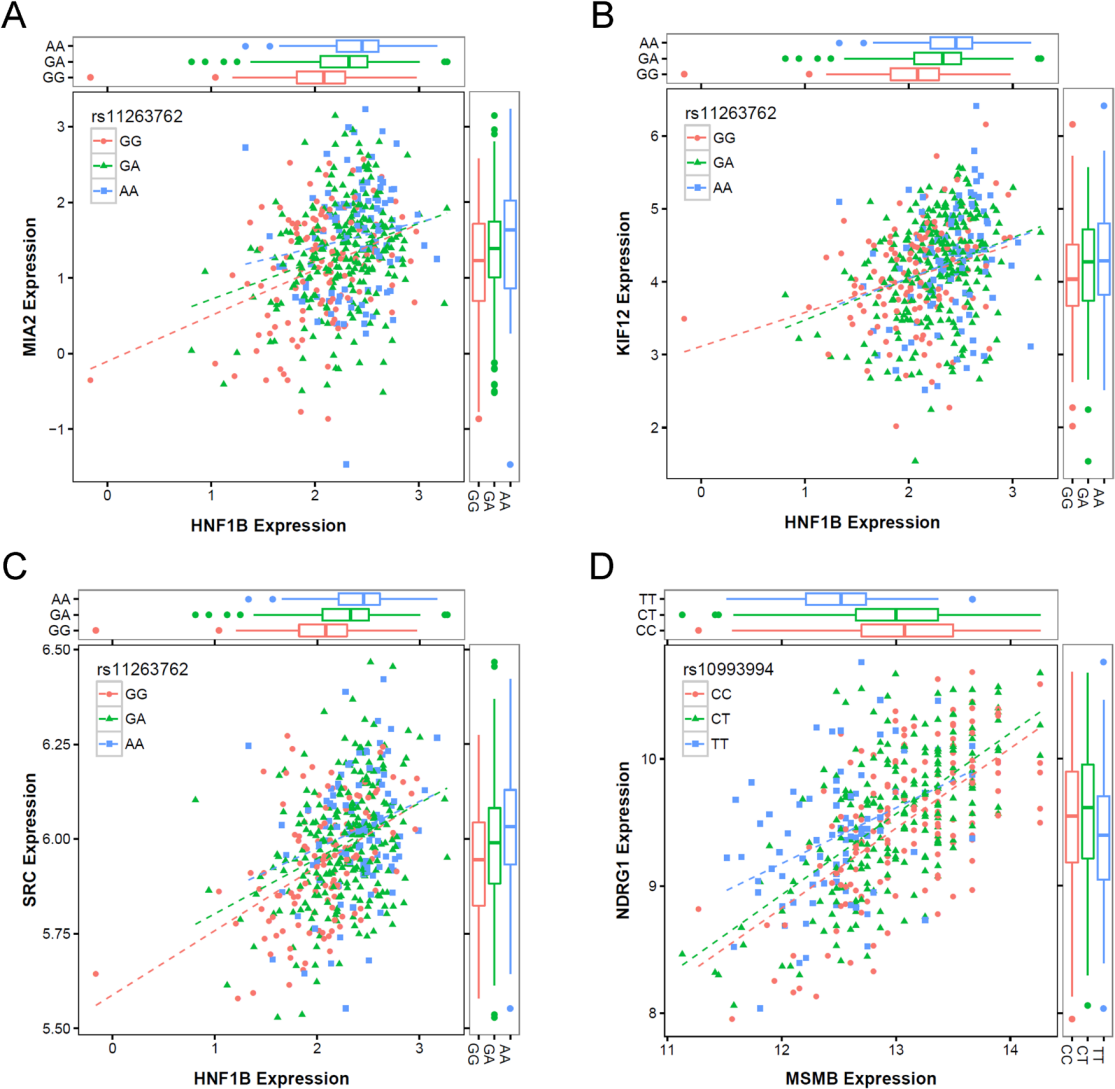
Co-expression scatterplots for four *cis*-mediator trios with evidence of replication in GTEx, including **(A)** rs11263762→*HNF1B*→*MIA2,* **(B)** rs11263762→*HNF1B* →*KIF12,* **(C)** rs11263762→*HNF1B*→*SRC,* and **(D)** rs10993994→*MSMB*→*NDRG1.* Each sub-figure presents the *cis*-gene expression on the horizontal axis and the trans-gene expression on the vertical axis, with individual points separated by the *cis*-eQTL genotype based on color and shape. Boxplots on the margins also summarize the expression distributions by eQTL genotype.

The latest release of Gene Tissue Expression (GTEx) project [30] currently has 87 prostate tissue samples with available genotype data. We queried the constituent *trans*-eQTL associations that corresponded to the *cis*-mediator relationships presented in Table 1 in the GTEx Portal to investigate whether consistent associations were observed in an independent dataset (Supplementary Table 2). For genes connected to *HNF1B*, we allowed any of 19 genotyped positions in LD with rs11263762 to be considered. Of the seven *trans*-associations reported, four corresponded to *trans*-eQTLs with p-values < 0.05 (rs4430796→*MIA2*, P = 6.5E-04; rs11263763→*KIF12*, P = 0.0044; rs10993994→*NDRG1*, P = 0.013; rs7405696→*SRC*, P = 0.038), with GTEx effect estimate directionality consistent with all seven discovery findings. Co-expression scatterplots for each of the four *cis*-mediator results with GTEx-replicated *trans*-eQTLs are presented in Figure 2.

Fine-mapping studies of the PRCA risk locus near *HNF1B* have indicated potentially multiple variants independently contributing to disease susceptibility for prostate [31] and endometrial cancers [32]. Thus, the peak *cis*-eQTL association may not completely capture downstream regulatory effects on potential HNF-1B targets. We subsequently relaxed the constraint of investigating *trans*-associations with peak *HNF1B cis*-SNP rs11263762 to all 33 potential PRCA risk *cis*-variants (Supplementary Table 3) for the nine *HNF1B*-connected gene nodes. Although the peak *trans*-eQTL SNP association varied for each gene, SNPs corresponding to significant or suggestive eQTLs were all included in a single LD block consisting of 20 SNPs (Supplementary Figure 2). The significance status of the *cis*-mediator relationship across the nine *trans* genes remained the same regardless of the selected SNP (peak *cis* or peak *trans*) within the LD block.

Previous studies investigating HNF-1B transcription factor targets in PRCA cell lines identified multiple potentially dysregulated genes relevant to PRCA. Hu et al. [33] discovered six putative HNF-1B target genes by leveraging publicly available microarray datasets and conducting HNF-1B transfection studies of multiple prostate cell lines. More recently, Ross-Adams et al. [34] discovered two separate genes (*FLRT3* and *SLC14A1*) that exhibited reduced expression in accordance with *HNF1B* over-expression in PC3. In our analyses, none of these genes exhibited compelling evidence of *trans*-eQTL effects with *HNF1B cis*-eQTLs consistent with HNF-1B regulation in normal prostate epithelium (min. *trans*-eQTL P = 0.009 across 272 tests; Supplementary Table 4).

*HNF1B* and *MSMB* also encode multiple isoforms, and differential isoform expression for these genes has been observed in comparisons of tumor and normal prostate tissues [35]. To explore whether significant eQTL SNPs corresponded to differential isoform usage, we conducted supplementary isoform-ratio QTL analyses using the R package *sQTLSeeker* [36] and isoform abundances derived from StringTie v1.2.4 [37]. We identified one intronic SNP, rs3110641, to be associated with differential isoform expression (P = 2.0E-04), although this SNP was not a significant *trans*-eQTL for any *HNF1B*-connected genes. Similarly, we did not observe any increase in mediation by any one particular *HNF1B* isoform, although the most highly expressed transcript (ENST00000225893) did account for the largest mediation effects across all significant *cis*-mediator trios and exhibited the strongest eQTL association with rs11263762 (P = 1.5E-05, Supplementary Figure 3). No alternative isoform transcripts were observed in sufficient quantity for *MSMB*.

It is possible due to measurement error or hidden confounders that the estimated undirected graph failed to include relevant *trans*-gene connections with significantly *cis*-regulated genes, and true co-expression relationships may have gone undetected. We conducted supplementary sensitivity analyses to identify all *trans*-eQTL associations for the 72 SNPs that were significant *cis*-eQTLs based on similar *cis*-mediator analyses agnostic of the expression network connections, yielding approximately 1.3 million tests. Using the same mediation p-value significance threshold defined above (P < 4.3E-05), only four additional trios were detected (Supplementary Table 5), none of which would have achieved significance by Bonferroni correction under this new multiple testing dimensionality (all P > 1E-07).

## DISCUSSION

It is now widely believed that a large proportion of genetic associations with complex traits are regulatory in nature, as trait-associated GWAS findings have been shown to be significantly enriched for eQTLs [38]. While multiple studies have investigated the potential regulatory roles of PRCA susceptibility loci on gene expression, they have largely focused on dysregulation of proximal genes. Thus, association findings only elucidate the initial functional consequences of these variants, which may in turn disrupt downstream molecular pathways. Using an expression network-directed analysis strategy, we identified evidence of three significant *cis*-mediator trios indicating potential downstream dysregulation of protein coding genes *SRC*, *NDRG1*, and *MIA2* by PRCA susceptibility loci in normal prostate epithelium, all of which are known to play prominent roles in tumorigenesis.

*HNF1B* encodes the protein hepatocyte nuclear factor-1 beta (HNF-1B), a transcription factor that plays a critical regulatory role in nephron and pancreas development [39]. HNF-1B and related protein HNF-1A comprise the HNF-1 sub-family of homeodomain-containing transcription factors, and may bind to DNA as homodimers or heterodimers [40]. Although primarily expressed in the liver, these transcription factors are known to regulate tissue-specific gene expression in the epithelia of a variety of organs [41], and may either activate or suppress transcription. Multiple cancer risk SNP associations have been reported at the *HNF1B* locus [31], although expression studies have produced conflicting results with respect to the regulatory effect of risk alleles on *HNF1B* in benign prostate tissue. These results have led to differing perspectives on the role of HNF-1B in progression of PRCA. For example, Griziano et al. [42] identified increased normal prostate *HNF1B* expression levels with the rs4430796-A allele across multiple ethnicities, and *HNF1B* knockdown in the LNCaP PRCA cell line resulted in reduced colony formation, proliferation, and viability. In contrast, Ross-Adams et al. [34] found no significant eQTL associations between four PRCA risk SNPs in normal prostate tissue samples; however, PRCA risk alleles for rs3760511 and rs11649743 corresponded to elevated *HNF1B* expression in tumor samples, with additional evidence indicating these variants are associated with reduced promoter methylation. In our analyses, risk-associated alleles exhibited patterns of upregulatory effects on *HNF1B* expression, suggesting oncogenic properties of *HNF1B* in the development of PRCA. Similar results were observed in the fine-mapping analysis of the *HNF1B* locus by Painter et al. [32] in endometrioid cancer, which identified the protective allele of SNP rs11263763 (r^2^ with rs11263762 = 0.61; *HNF1B cis*-eQTL P = 4.8E-12) to be associated with reduced *HNF1B* promoter activity. Recent analysis of *HNF1B* in breast cancer has also indicated *HNF1B* overexpression induces transformation and epithelial-to-mesenchymal transition in the NMuMG epithelial cell-line [43], providing further evidence of an oncogenic role of *HNF1B* in cancers of epithelial origin.

*HNF1B* corresponded to four out of seven of the reported *cis*-mediator associations we identified, indicating potential dysregulation of multiple HNF-1B transcription factor targets by PRCA susceptibility variants near *HNF1B*. *SRC* encodes the proto-oncogene c-Src, a member of the Src family kinases [44], and Src pathways play a prominent role in PRCA tumorigenesis [45, 46]. *SRC* expression has also been shown to be directly regulated by HNF-1A (although not HNF-1B) via an alternative tissue-specific HNF-1 promoter in multiple cell-types [47]. *MIA2* encodes the melanoma-inhibitory activity 2 (MIA2) protein, which belongs to the MIA gene family, and is similarly transcriptionally regulated by HNF-1A [48]. Although corresponding to tumor suppressive properties in hepatocellular carcinoma [49], *MIA2* exhibits protumoral properties in oral squamous cell carcinoma, demonstrating increases in invasion, survival, and angiogenesis [50]. HNF-1A induced expression of *MIA2* has also been implicated in pancreatic cancer [51]. Additionally, expression of *KIF12* has been shown to be directly regulated by HNF-1B in kidneys [52]. While *KIF12* has been implicated as a disease severity modifier of renal cystic disease via HNF-1B-induced transcription [53], its potential role in PRCA etiology is not immediately clear. Eight previously identified PRCA HNF-1B target genes were not replicated in our analyses; however, it is important to note these genes were validated in the correspondent studies based on HNF-1B transfected PC3 PRCA cell-line experiments, not normal prostate epithelial cells.

*MSMB* encodes the prostate secretory protein 94 (PSP94), which is predominantly expressed in prostate. Reduced or lost *MSMB* expression is commonly observed in PRCA tumors [54, 55] and is generally associated with poor prognosis and increased risk of recurrence [56, 57], although other studies have produced contrary findings [58]. Suppression of *MSMB* in prostate epithelial cells also promotes anchorage-independent growth [59]. Multiple genetic association studies [60–62] have replicated the correspondent eQTL variant, rs10993994, with PRCA susceptibility, and the risk-associated T allele has been shown to result in reduced expression of PSP94 [63, 64]. Consequently, PSP94 is widely believed to confer protective effects against PRCA tumorigenesis, although the underlying mechanism is poorly understood. It has been postulated PSP94 may act as a tumor suppressor or limit fungal pathogenic infection of prostate tissue [65]. In our analyses, we identified a *trans*-assocition of *NDRG1* expression with PRCA risk SNP rs10993994 to be mediated by *MSMB* expression, with the two genes exhibiting positively correlated co-expression patterns. Although the potential biological mechanisms linking *MSMB* expression to dysregulation of *NDRG1* are not immediately clear, it is hypothesized that PSP94 peptides may activate signal transduction pathways relevant to apoptosis via cell surface receptors [66]. *NDRG1* encodes the N-myc downstream regulated gene 1 (NDRG1) protein, and *NDRG1* gene expression is repressed by N-myc and c-myc [67]. The NDRG1 protein participates in multiple cancer -related pathways, and P53-induced expression of *NDRG1* has been shown to suppress cell growth and proliferation [68]. NDRG1 has also been shown to inhibit activation of c-Src by preventing protein-protein interactions between c-Src and EGFR [69]. Specific to PRCA, NDRG1 expression is inversely associated with Gleason score [70], and immunohistochemistry experiments have indicated reduced NDRG1 expression in neoplastic tissue compared to adjacent normal cells [71]. Thus, *MSMB* may confer protective effects against PRCA via signaling cascades that upregulate expression of *NDRG1*.

There are a number of limitations to our study that warrant mention. First, our network inference is based solely on mRNA expression, and only captures a fraction of the biomolecular intermediaries of causal pathways impacted by dysregulation of the *cis*-associated genes. The relatively small sample size in comparison to the large gene count may also have limited our ability to estimate the graphical network structure, and smaller partial correlations may have gone undetected. Second, other types of RNA beyond protein-coding transcripts are also known to possess regulatory effects, including long non-coding RNAs and miRNAs. For example, HNF-1B was recently identified to regulate the miR-200 cluster in renal cells [72]. Integrative analyses of the co-expression patterns between the variety of RNA species may provide a more complete perspective on the impact of PRCA susceptibility genetics on the prostate transcriptome, although these likely necessitate larger datasets. Third, rigorous replication and lab validation of our findings will be necessary to verify the regulatory associations we have identified.

By integrating gene co-expression patterns and causal mediation analyses in the evaluation of transcriptional dysregulation by PRCA risk loci, we identified multiple plausible downstream effects mediated by PRCA risk genes *MSMB* and *HNF1B.* Our work provides the foundation for novel hypotheses for further investigation into the functional genetics of PRCA susceptibility and tumor progression.

## MATERIALS AND METHODS

### Study samples

All analyses were conducted on a normal prostate tissue eQTL dataset comprised of 471 samples that passed strict quality control criteria (dbGap accession phs000985.v1.p1), previously detailed elsewhere [17, 73]. Briefly, normal prostate tissue was acquired from an archive collection of fresh frozen material obtained from patients with either radical prostatectomy or cystoprostatectomy, which was reviewed to identify samples that met the following criteria: absence of prostate tumor, Gleason score was <7 for the presenting tumor, absence of high-grade prostatic intraepithelial neoplasia and benign prostatic hyperplasia, normal prostatic epithelial glands representing ≥ 40% of all cells, lymphocytic population representing ≤ 2% of all cells, and the normal epithelium was from the posterior region of the prostate. Informed consent was obtained from all subjects and the study was approved by the Mayo Clinic Institutional Review Board.

### Genotyping and imputation

DNA was extracted using the Puregene tissue extraction protocol per the manufacturer’s recommendations and DNA quality was assessed by examining 260/280 ratio and DNA yield. Samples were genotyped using Illumina Infinium 2.5M bead arrays based on the manufacturer’s protocol (Illumina, San Diego, CA, USA). Standard quality control analyses were performed to identify poor-quality samples or SNPs. Untyped SNPs as well as missing genotypes for typed SNPs were imputed using SHAPEIT [74] and IMPUTE2 [75] with reference files from the 1000 Genomes Phase I integrated variant set.

### RNA sequencing and expression quantification

RNA was extracted using the QIAGEN miRNeasy Mini Kit and the QIAcube instrument in accordance with the manufacturer’s instructions, and RNA quality was assessed by evaluating the RNA integrity number (>7) and the 260/280 ratio. RNA libraries were prepared using the TruSeq RNA Sample Prep Kit v2 (Illumina, San Diego, CA, USA) according to the manufacturer’s instructions. Paired-end sequencing was performed on an Illumina HiSeq 2000 using TruSeq SBS sequencing kit version 3 and HCS v2.0.12 data collection software. A minimum of 50 million total reads per sample was required for analysis; samples with insufficient reads were re-sequenced and resultant BAM files were merged.

RNA-seq data were analyzed with the use of the MAP-R-Seq pipeline [76]. Paired-end reads were aligned by TopHat 2.0.6 [77] against the hg19 genome build using the bowtie1 aligner option. RSeQC [78] was used to calculate various quality control metrics to identify problematic samples. Such metrics include: the genomic distance between paired-end reads, the sequencing depth for predicted alternate splicing events, the rate of duplicate reads, and the evenness of each sample’s gene body coverage. Gene counts were quantified for 102,279 RNA features based on the ENSEMBL GRCh37.75 gene definition file, of which 17,216 were identified as protein coding genes from the biotype annotation field and declared to be expressed based on a median gene read count ≥ 10.

To remove potential biases such as GC content and differences in sequencing depth, gene read counts were normalized using conditional quantile normalization [79]. To account for latent sources of non-genetic variation in gene expression, we applied principal components analysis (PCA) to the complete normalized gene expression matrix, identifying 13 PCs for inclusion as covariates in the eQTL analysis, each accounting for ≥1% of the total variation.

### eQTL analyses

For the 202 previously reported PRCA risk SNPs (Supplementary Table 1), we declared all observed and high-quality imputed variants (allelic *r*^2^ > 0.3) within 200kb and with linkage disequilibrium (LD) *r*^2^>0.5 eligible for eQTL analysis as candidate risk variants, resulting in a total of 8073 variants of interest. All eQTL association analyses were conducted using the MatrixEQTL R package [80], adjusting for PE, PL, and the top 13 expression PCs. Associations were defined as *cis* if the eQTL SNP was within 1 Mb of the transcript. To avoid spurious associations due to long-range LD patterns, we declared all transcripts at least 10 Mb from a *cis*-gene to be eligible as *cis*-mediated *trans*-genes.

### Network inference

To identify a large subset of potentially relevant PRCA susceptibility genes for network inference, we conducted an initial transcriptome-wide eQTL screening for expressed transcripts with all tag PRCA risk SNPs under liberal selection criteria. The rationale for this strategy was the notion that risk SNP associations would propagate through relevant co-expression networks. The smallest eQTL association p-value per transcript across all tested SNPs was Bonferroni-adjusted for the number of original risk loci (i.e., 202), and all transcripts corresponding to a FDR < 0.20 were selected for network analysis. This permissive significance threshold accommodated efficient dimensionality reduction while limiting the potential exclusion of false negative results. We estimated an undirected graph using the Meinshausen-Buhlmann method as part of the *huge* R package [81]. Default settings were used for all regularization parameters.

### Cis-Mediators and causal inference

**Figure 3:**
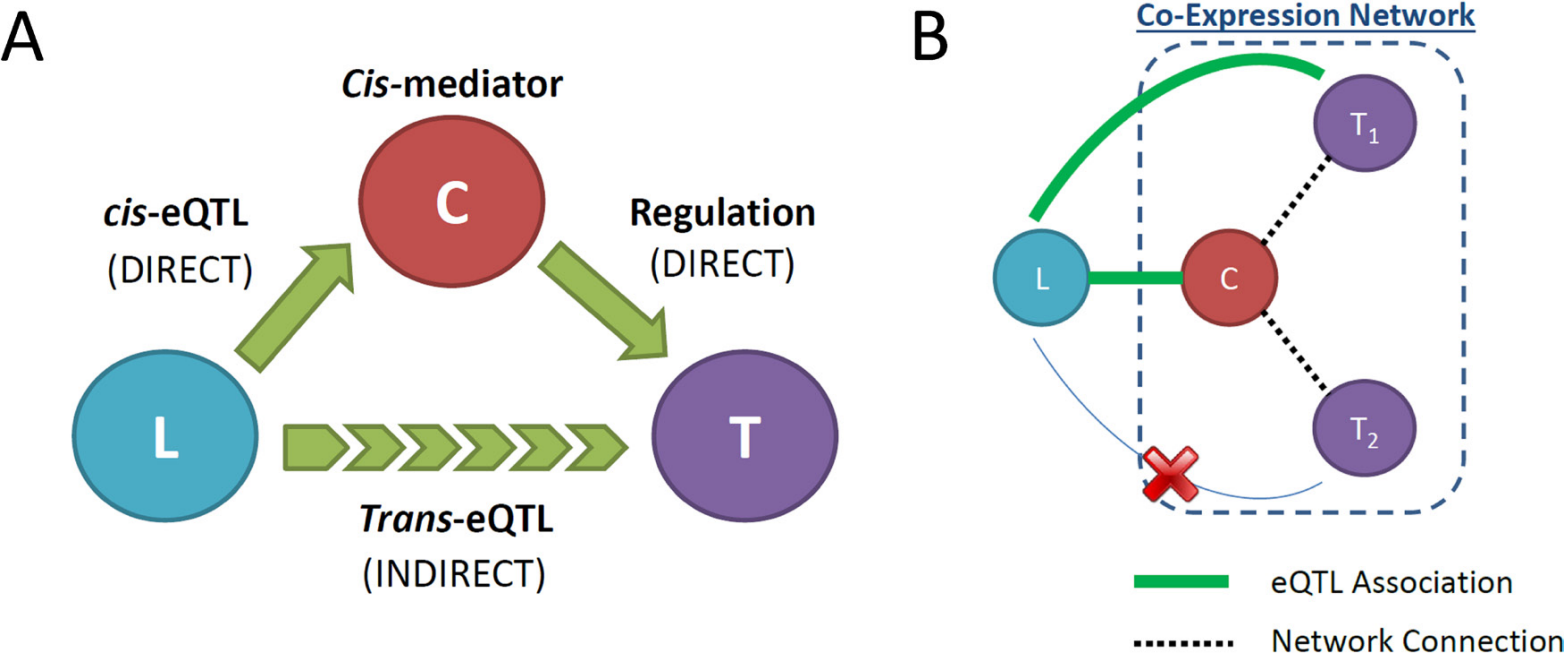
**(A)** Diagram indicating the causal relationships among the *cis*-eQTL variant, *L,* the cis-eQTL gene, *C,* and the trans-eQTL gene, *T*, that define the *cis*-mediator causal pathway. The trans-eQTL association is an indirect relationship mediated by the *cis*-regulated gene *C*. **(B)** Illustration of co-expression network strategy for identifying potential *cis*-mediated trans-eQTLs. Solid green lines indicate significant eQTL association, while dotted black lines indicate co-expression network connections between node genes. Here, locus *L* demonstrates a shared eQTL association with gene *C* and network-connected gene *T*_*1*_(but not *T*_*2*_), which can be further investigated using causal mediation analysis.

A *cis*-mediator causal relationship is comprised of the eQTL variant, denoted *L*, the *cis*-regulated transcript (or *cis*-gene), denoted *C*, and the *trans*-regulated transcript (or *trans*-gene), denoted *T*. Thus, the eQTL variant, *cis*-gene, and *trans*-gene comprise a candidate *cis*-mediator trio (*L*, *C*, *T*), where the causal relationship can be characterized as *L* → *C* → *T* with arrows indicating causal direction (Figure 3A). The peak *cis*-eQTL SNP per gene was defined according to the smallest eQTL association p-value. All peak *cis*-eQTL associations with a p-value below a Bonferroni-adjusted threshold for all *cis*-eQTL association tests (P < 0.05/144,628 = 3.5E-07) were considered to be significant. All mRNAs connected to significant *cis*-genes in the expression network and in *trans* with the corresponding peak *cis*-eQTL variant were declared to be eligible *cis*-mediator trios (Figure 3B).

Causal inference was conducted for all eligible (*L*, *C*, *T*) trios to investigate whether *trans*-gene associations with *cis*-SNPs were mediated by the corresponding *cis*-gene expression. We employed model-based causal inference analysis using the *cit* R package [82, 83]. The mediation analysis methods in *cit* correspond to a conservative omnibus intersection-union test for four constituent association relationships that comprise the causal mediation relationship *L* → *C* → *T*, returning the maximum p-value across these tests. We additionally quantified mediation by calculating the proportion of the *trans*-eQTL effect estimate β^T attenuated by the additional adjustment of the corresponding *cis*-gene expression values as a covariate. For *cis*-gene adjusted *trans*-eQTL effect ${\hat{\beta }}_{T}^{adj}$ we define the estimated mediated proportion as $M=\frac{{\hat{\beta }}_{T}-{\hat{\beta }}_{T}^{adj}}{{\hat{\beta }}_{T}}$. All mediation tests with p-values below a Bonferroni-adjusted *α*-level of 0.05 were reported as significant *cis*-mediated *trans*-eQTLs.

## SUPPLEMENTARY MATERIALS FIGURES AND TABLES







## Abbreviations

eQTL: expression quantitative trait locus
FDR: false discovery rate
GTEx: Gene Tissue Expression project
GWAS: genome-wide association study
LD: linkage disequilibrium
PCA: principal components analysis
PRCA: prostate cancer
SNP: single-nucleotide polymorphism
