# Supplementary material for: Network-directed cis-mediator analysis of normal prostate tissue expression profiles reveals downstream regulatory associations of prostate cancer susceptibility loci
Source: Oncotarget. 2017 Sep 8;8(49):85896–908. doi: 10.18632/oncotarget.20717 (PMC5689655; doi:10.18632/oncotarget.20717)
Supplement: Supplementary file 1 [file oncotarget-08-85896-s001.pdf]

# Network-directed cis-mediator analysis of normal prostate tissue expression profiles reveals downstream regulatory associations of prostate cancer susceptibility loci

## SUPPLEMENTARY MATERIALS

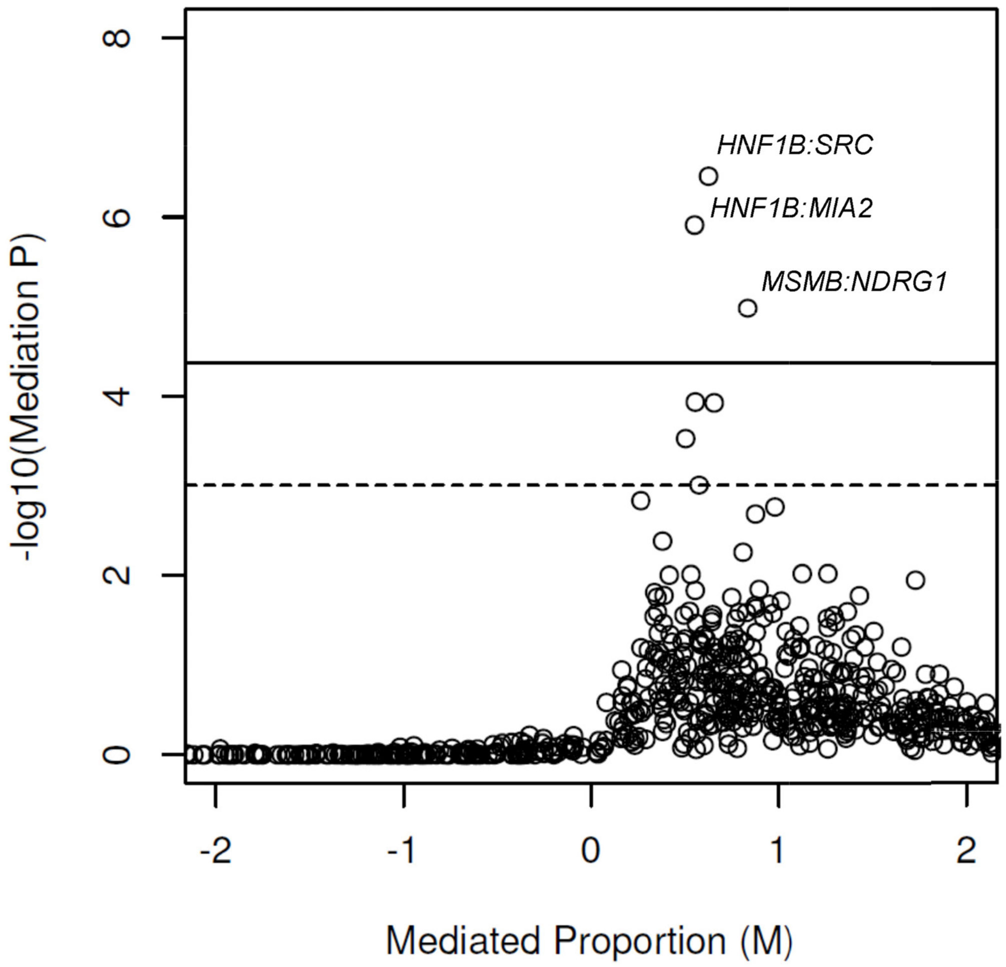


**Supplementary Figure 1: Scatterplot of mediation effect proportion** $M = \frac{\hat{\beta}_T - \hat{\beta}_T^{adj}}{\hat{\beta}_T}$ **(horizontal axis) and corresponding mediation p-values (vertical axis) for all 1168 candidate *cis*-mediator trios.** Horizontal lines indicated significant (solid) and suggestive (dashed) thresholds, with significant results labeled by the corresponding *cis*-gene *trans*-gene pair (*C:T*). Values outside of the range of [0, 1] are possible due to noise in estimates of $\beta_T$ and $\beta_T^{adj}$ .

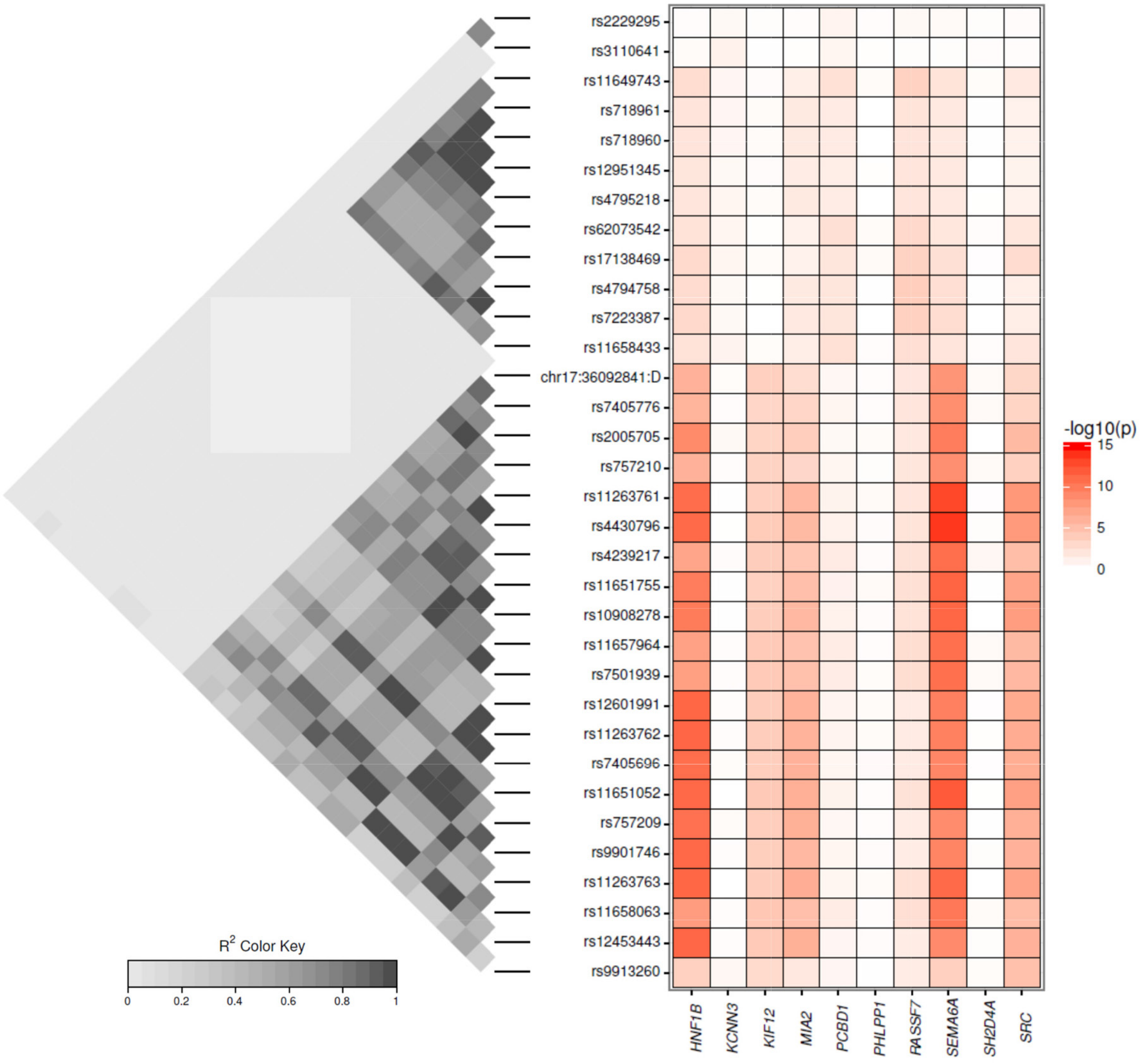


**Supplementary Figure 2: Tile-plot indicating eQTL associations for *HNF1B* and nine neighbor node genes in the co-expression network for variants in LD with PRCA risk loci proximal to *HNF1B*.** The left figure indicates LD ($R^2$) between the SNPs, while the tileplot to the right displays the eQTL association results for each SNP-gene pair, with the gradient from white to red corresponding to –log10(p-values).

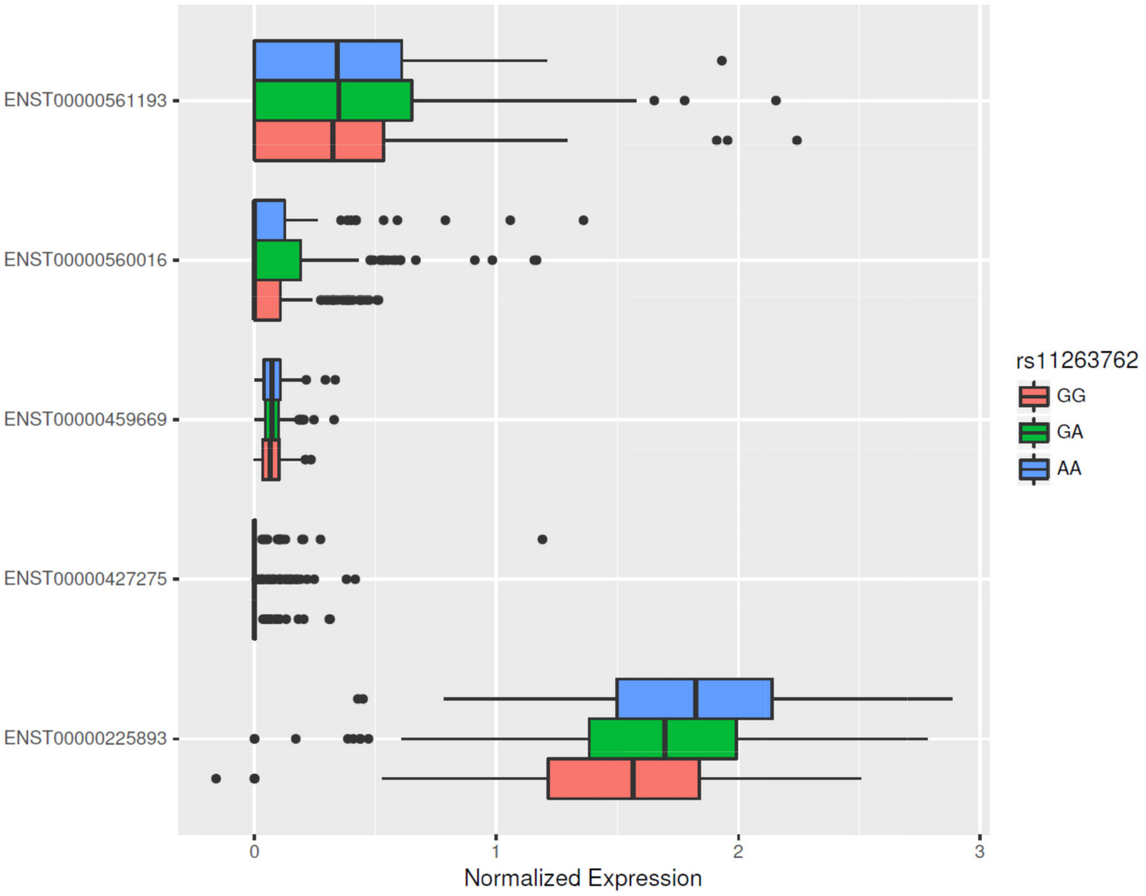


**Supplementary Figure 3: Expression of detected *HNF1B* isoforms by peak cis-eQTL SNP rs11263762 genotype.** Isoform-specific expression was computed by multiplying isoform-abundance ratios from StringTie to normalized total expression values.

**Supplementary Table 1: Significant PRCA risk loci *cis*-eQTL associations applied in *cis*-mediator analyses**

See Supplementary File 1

**Supplementary Table 2: Discovery cohort and GTEx prostate tissue *trans*-eQTL association results for seven significant or suggestive *cis*-mediator trios**

| | | | | Discovery Results | | GTEx Results** | |
|---|---|---|---|---|---|---|---|
| ***Cis-Gene*** | ***Trans-Gene*** | **rsID*** | **Ref/Alt** | ***β*** | **P** | ***β*** | **P** |
| *HNF1B* | *MIA2* | rs4430796 | A/G | -0.20 | 4.2E-06 | -0.45 | 6.5E-04 |
| *HNF1B* | *KIF12* | rs11263763 | A/G | -0.13 | 1.1E-04 | -0.29 | 0.0044 |
| *MSMB* | *NDRG1* | rs10993994 | C/T | -0.10 | 1.0E-05 | -0.23 | 0.013 |
| *HNF1B* | *SRC* | rs7405696 | G/C | 0.04 | 4.6E-07 | 0.22 | 0.038 |
| *HNF1B* | *SEMA6A* | rs757209 | G/A | 0.13 | 1.6E-09 | 0.14 | 0.26 |
| *AS3MT* | *TMEM121* | rs11191385 | G/T | -0.08 | 3.5E-05 | -0.13 | 0.31 |
| *TECPR1* | *UBA5* | rs6958572 | G/A | 0.02 | 4.1E-04 | 0.05 | 0.61 |

Results are sorted by GTEx *trans*-eQTL p-value.
*For *MIA2*, *KIF12*, *SRC*, and *SEMA6A*, the SNP with the minimum p-value across the 19 genotyped SNPs in the rs11263762 LD block is reported.
**Effect estimate sign flipped if necessary to be consistent with Ref/Alt allele designations in discovery set.

**Supplementary Table 3: *Cis*-eQTL association results for all 33 PRCA risk loci variants tested for *HNF1B* expression, sorted by chromosome 17 position**

| rsID | Chr17 Pos. | Major | Minor | Freq | *β* | *P* |
|---|---|---|---|---|---|---|
| rs2229295 | 36047276 | G | T | 0.171725 | -0.01499 | 0.60 |
| rs3110641 | 36047417 | G | A | 0.226837 | -0.01879 | 0.47 |
| rs11649743 | 36074979 | G | A | 0.21246 | -0.08408 | 0.0022 |
| rs718961 | 36077099 | G | A | 0.244282 | -0.06965 | 0.0079 |
| rs718960 | 36077279 | C | T | 0.244333 | -0.06949 | 0.0081 |
| rs12951345 | 36077863 | A | C | 0.253725 | -0.06667 | 0.010 |
| rs4795218 | 36078510 | G | A | 0.244726 | -0.06839 | 0.0092 |
| rs62073542 | 36079565 | G | A | 0.213671 | -0.0778 | 0.0053 |
| rs17138469 | 36080165 | G | C | 0.196365 | -0.09324 | 0.0013 |
| rs4794758 | 36080428 | C | T | 0.277201 | -0.07895 | 0.0016 |
| rs7223387 | 36082473 | T | G | 0.280351 | -0.08211 | 9.86E-04 |
| rs11658433 | 36082907 | A | C | 0.223341 | -0.07668 | 0.0064 |
| chr17:36092841:D | 36092841 | G | GT | 0.372605 | -0.11461 | 1.04E-06 |
| rs7405776 | 36093022 | G | A | 0.345847 | -0.1087 | 1.47E-06 |
| rs2005705 | 36096300 | G | A | 0.408634 | -0.13479 | 1.24E-09 |
| rs757210 | 36096515 | C | T | 0.338658 | -0.11032 | 1.06E-06 |
| rs11263761 | 36097775 | A | G | 0.440133 | -0.15124 | 1.22E-11 |
| rs4430796 | 36098040 | A | G | 0.429712 | -0.15087 | 8.67E-12 |
| rs4239217 | 36098987 | A | G | 0.359121 | -0.12065 | 1.04E-07 |
| rs11651755 | 36099840 | T | C | 0.427316 | -0.14407 | 1.37E-10 |
| rs10908278 | 36099952 | A | T | 0.426813 | -0.14478 | 1.19E-10 |
| rs11657964 | 36100767 | G | A | 0.351645 | -0.12402 | 4.78E-08 |
| rs7501939 | 36101156 | C | T | 0.351438 | -0.1243 | 4.09E-08 |
| rs12601991 | 36101633 | G | T | 0.467734 | 0.154977 | 4.80E-12 |
| rs11263762 | 36101926 | G | A | 0.466454 | 0.154849 | 4.36E-12 |
| rs7405696 | 36102035 | G | C | 0.467252 | 0.15168 | 1.33E-11 |
| rs11651052 | 36102381 | G | A | 0.423561 | -0.15429 | 6.93E-12 |
| rs757209 | 36102833 | G | A | 0.452149 | 0.150165 | 2.32E-11 |
| rs9901746 | 36103149 | G | A | 0.466766 | 0.155479 | 6.58E-12 |
| rs11263763 | 36103565 | A | G | 0.425414 | -0.15584 | 4.81E-12 |
| rs11658063 | 36103872 | G | C | 0.352376 | -0.12894 | 2.10E-08 |
| rs12453443 | 36104121 | C | G | 0.462849 | 0.157806 | 5.01E-12 |
| rs9913260 | 36105897 | G | A | 0.232428 | -0.09241 | 0.00023 |

**Supplementary Table 4: *Trans*-eQTL results for previously identified putative HNF-1B target genes in PRCA cell line studies and *HNF1B cis*-eQTL variants**

| Reference | Peak *Trans* SNP | | *Trans*-eQTL | | HNF1B *cis*-eQTL | |
|---|---|---|---|---|---|---|
| | Gene | rsID | Beta | P | Beta | P |
| *Hu et al.* | *BAG1* | rs12453443 | -0.02 | 0.070 | 0.16 | 5.0E-12 |
| | *ERBB4* | rs7405776 | 0.05 | 0.070 | -0.11 | 1.5E-06 |
| | *ESR1* | rs3110641 | -0.09 | 0.012 | -0.02 | 0.47 |
| | *HSPD1* | rs4430796 | 0.02 | 0.11 | -0.15 | 8.7E-12 |
| | *NR4A1* | rs11263761 | -0.03 | 0.34 | -0.15 | 1.2E-11 |
| | *PIK3CG* | rs9913260 | 0.05 | 0.0086 | -0.09 | 2.3E-04 |
| *Ross-Adams et al.* | *FL3RT* | rs11649743 | -0.06 | 0.051 | -0.08 | 2.2E-03 |
| | *SLC14A1* | rs2005705 | -0.04 | 0.12 | -0.13 | 1.2E-09 |

**Supplementary Table 5: Additional suggestive (P<1E-04) *cis*-mediated *trans*-eQTL associations agnostic of the gene co-expression network**

See Supplementary File 2
