# Supplementary material for: Network-directed cis-mediator analysis of normal prostate tissue expression profiles reveals downstream regulatory associations of prostate cancer susceptibility loci
Source: Oncotarget. 2017 Sep 8;8(49):85896–908. doi: 10.18632/oncotarget.20717 (PMC5689655; doi:10.18632/oncotarget.20717)
Supplement: Supplementary file 2 [file oncotarget-08-85896-s002.pdf]

Supplementary Table 1: Significant PRCA risk loci *cis*-eQTL associations applied in *cis*-mediator analyses.

rsID	Chr	Pos	Major	Minor	Gene	Chr	Start	Stop	β	P
rs5945624	23	51252928	A	G	NUDT11	23	51232863	51239448	0.47	1.16E-132
rs113645266	6	153437735	T	C	RGS17	6	153325594	153452384	-0.63	3.00E-103
rs12655062	5	1890877	G	A	IRX4	5	1877541	1887350	-1.19	2.03E-102
rs12976534	19	38743962	A	G	PPP1R14A	19	38741877	38747231	-0.34	9.35E-88
rs10993994	10	51549496	C	T	NCOA4	10	51565108	51590734	0.35	1.07E-78
rs461251	17	619162	A	G	FAM57A	17	635652	646206	-0.24	4.27E-63
chr6:160581543:I	6	160581543	T	TG	SLC22A3	6	160769300	160876014	-0.67	1.59E-51
rs4830661	23	9807693	G	A	SHROOM2	23	9754496	9917483	0.14	3.83E-48
rs12622106	2	20883561	C	T	C2orf43	2	20883788	21022882	-0.33	1.75E-45
rs4962720	10	126696840	G	T	CTBP2	10	126676421	126849739	0.14	8.43E-39
rs58057291	2	238395479	T	A	MLPH	2	238394071	238463961	-0.20	2.91E-38
rs437948	17	623356	C	G	VPS53	17	411908	624957	-0.10	3.02E-38
rs708723	1	205739266	T	C	RAB7L1	1	205737114	205744588	-0.19	6.77E-38
rs10993994	10	51549496	C	T	MSMB	10	51549498	51562517	-0.32	7.42E-38
rs5937025	23	70142030	T	C	SLC7A3	23	70145432	70150975	0.42	1.53E-35
rs11191385	10	104513049	G	T	C10orf32-ASMT	10	104614029	104661656	-0.15	6.55E-35
rs4830661	23	9807693	G	A	GPR143	23	9693386	9754337	0.17	4.59E-33
rs11191385	10	104513049	G	T	AS3MT	10	104629273	104661656	-0.22	1.03E-32
rs145076668	6	31188436	C	T	HCG27	6	31165537	31171745	0.46	1.07E-31
rs3135392	6	32409242	C	A	HLA-DRB5	6	32485120	32498064	-0.51	4.10E-31
rs41298814	22	19750773	T	C	TBX1	22	19744226	19771116	0.66	8.16E-31
rs2474694	17	618039	G	A	GEMIN4	17	647654	657239	-0.12	1.06E-30
rs11158289	14	61032390	A	G	C14orf39	14	60863187	60982261	-0.46	2.22E-28
rs9380150	6	30010492	T	C	HLA-G	6	29794744	29798902	0.87	7.88E-28
rs114376585	6	32192331	G	A	NOTCH4	6	32162620	32191844	0.17	3.89E-27
rs11191385	10	104513049	G	T	C10orf32	10	104613980	104624718	-0.10	1.98E-25
rs747181	23	70432708	G	A	GJB1	23	70435044	70445366	-0.21	4.75E-25
rs2356575	6	109367255	T	C	SESN1	6	109307640	109416022	0.20	1.82E-24
chr6:160581543:I	6	160581543	T	TG	SLC22A1	6	160542821	160579750	-0.44	3.12E-24
rs72626278	19	54793239	G	A	LILRA3	19	54799854	54809952	-0.68	3.30E-24
rs12639887	4	95505975	C	T	BMPR1B	4	95679119	96079599	-0.19	7.03E-24
rs7567304	2	10790029	C	T	NOL10	2	10710892	10830101	0.06	2.85E-23
rs3129878	6	32408735	A	C	HLA-DQA1	6	32595956	32614839	0.40	4.34E-20
rs2891600	19	38767110	T	C	SPINT2	19	38734675	38783254	0.08	2.21E-19
rs6958572	7	97789351	G	A	BHLHA15	7	97840739	97842291	0.25	2.62E-18
rs79618460	2	238419959	T	C	RAB17	2	238482965	238510257	-0.27	6.54E-18
rs144721865	6	31156041	C	T	HLA-C	6	31236526	31239907	-0.22	9.44E-18
rs11568818	11	102401661	T	C	MMP7	11	102391239	102401484	-0.59	1.22E-17
rs12262998	10	104428716	C	T	TMEM180	10	104221149	104236802	-0.21	7.92E-17
rs3105751	6	160822675	A	G	SLC22A2	6	160592093	160698670	-0.34	2.81E-16
rs9306895	2	20878153	T	C	GDF7	2	20866424	20873418	-0.20	2.89E-16
rs2891600	19	38767110	T	C	CTB-102L5.4	19	38779791	38795550	0.09	3.85E-16
rs3771578	2	242391212	C	T	SEPT2	2	242254515	242293442	0.08	6.48E-16
rs75056606	1	150718602	T	G	CTSS	1	150702672	150738433	-0.18	5.77E-14
rs61847060	10	51510203	G	A	AGAP7	10	51464162	51486327	0.29	1.02E-13
rs61436251	3	170083629	C	G	SKIL	3	170075466	170114623	-0.13	1.17E-13

chr6:160581543:I	6	160581543	T	TG	PLG	6	161123270	161174347	-0.28	1.40E-13
rs6958572	7	97789351	G	A	TECPR1	7	97843936	97881563	0.08	3.78E-13
rs743862	6	32381939	T	C	RNF5	6	32146131	32151930	-0.05	5.36E-13
rs2506901	1	10563492	A	G	APITD1	1	10490159	10512210	0.06	5.57E-13
rs12493488	3	87209236	G	A	CHMP2B	3	87276421	87304698	0.06	7.02E-13
rs7642163	3	113223410	T	C	WDR52	3	113005777	113160457	-0.06	9.62E-13
rs3129878	6	32408735	A	C	HLA-DQB2	6	32723875	32731311	-0.36	9.86E-13
rs9267853	6	32195251	C	T	HLA-DQB1	6	32627244	32636160	0.43	1.58E-12
rs1983891	6	41536427	C	T	FOXP4	6	41514164	41570122	-0.09	1.96E-12
rs11263762	17	36101926	G	A	HNF1B	17	36046435	36105237	0.15	4.36E-12
rs75056606	1	150718602	T	G	SV2A	1	149874870	149889434	-0.15	1.22E-11
rs1746816	9	110145326	C	A	RAD23B	9	110045418	110094475	-0.04	1.36E-11
rs12419367	11	2217280	G	A	ASCL2	11	2289725	2292182	0.21	2.39E-11
rs3798439	6	76566734	G	T	MYO6	6	76458909	76629254	0.09	2.64E-11
rs34582151	19	38750100	G	C	CATSPERG	19	38826415	38861589	-0.11	3.29E-11
rs9979125	21	42907168	C	T	TMPRSS2	21	42836478	42903043	0.15	7.30E-11
rs4899359	14	71027964	A	G	SYNJ2BP	14	70838148	70883778	0.04	8.09E-11
rs17122571	12	48421034	A	G	COL2A1	12	48366748	48398269	1.26	1.74E-10
rs111366118	6	30075903	C	T	VARS2	6	30876019	30894236	0.08	1.79E-10
rs145076668	6	31188436	C	T	POU5F1	6	31132119	31148508	0.29	2.60E-10
rs143466021	6	32443223	G	A	VWA7	6	31733367	31745108	-0.15	6.04E-10
rs7591769	2	173404886	G	A	ITGA6	2	173292082	173371181	-0.08	1.33E-09
rs6062509	20	62362563	T	G	LIME1	20	62366815	62370456	0.07	1.98E-09
rs79618460	2	238419959	T	C	AC104667.3	2	238499663	238504624	-0.29	4.56E-09
rs116483198	6	31145116	C	T	PSORS1C1	6	31082527	31107869	0.29	5.52E-09
rs3129878	6	32408735	A	C	HLA-DQA2	6	32709119	32714992	-0.45	1.87E-08
rs11114197	12	80024095	T	A	PAWR	12	79968759	80084877	-0.05	1.95E-08
rs115688463	6	31199573	C	T	MICA	6	31371356	31383092	0.19	2.00E-08
rs1632108	1	10561923	C	T	APITD1-CORT	1	10490159	10512210	0.06	2.13E-08
rs6977321	7	97774859	G	A	LMTK2	7	97736197	97838945	0.05	3.03E-08
rs3129878	6	32408735	A	C	HLA-DRB1	6	32546546	32557625	0.18	3.89E-08
rs1048665	20	62331989	T	G	ZGPAT	20	62338817	62367494	-0.04	4.61E-08
rs1755594	1	10565488	T	C	DFFA	1	10516579	10532583	-0.03	5.76E-08
rs12799145	11	58940435	A	G	DTX4	11	58938903	58976060	0.12	6.42E-08
rs11240554	1	205671644	C	T	NUCKS1	1	205681947	205719404	0.03	7.44E-08
rs78239819	12	48412550	C	T	TMEM106C	12	48357352	48362661	0.11	9.63E-08
chr2:242355534:D	2	242355534	CTG	C	HDLBP	2	242166679	242256476	0.04	1.41E-07
rs7632169	3	128114587	T	C	RUVBL1	3	127783621	127872757	0.04	1.49E-07
rs10411731	19	42014057	T	A	CEACAM21	19	42055886	42093197	-0.17	1.61E-07
rs785373	5	115655585	G	C	COMMD10	5	115420688	115748459	0.04	3.41E-07
