# Supplementary material for: Network-directed cis-mediator analysis of normal prostate tissue expression profiles reveals downstream regulatory associations of prostate cancer susceptibility loci
Source: Oncotarget. 2017 Sep 8;8(49):85896–908. doi: 10.18632/oncotarget.20717 (PMC5689655; doi:10.18632/oncotarget.20717)
Supplement: Supplementary file 3 [file oncotarget-08-85896-s003.pdf]

Supplementary Table 5: Additional suggestive (P<1E-04) *cis*-mediated *trans*-eQTL associations agnostic of the gene co-expression network.

eQTL Variant			*Cis*-Gene						*Trans*-Gene						Mediation		
rsID	Chr:pos (hg19)	Alleles[a]	Gene	Chr	Start	Stop	βC	PC	Gene	Chr	Start	Stop	βT	PT	P	$\beta Tadj$	M
rs10993994	10:51549496	C/T	*MSMB*	10	51549498	51562517	-0.32	7.4E-38	*LDLRAD4*	18	13217497	13652754	-0.10	7.8E-09	1.5E-07	-0.04	0.58
rs10993994	10:51549496	C/T	*MSMB*	10	51549498	51562517	-0.32	7.4E-38	*SLC27A2*	15	50474393	50528592	0.10	4.0E-05	4.0E-05	0.03	0.72
rs10993994	10:51549496	C/T	*MSMB*	10	51549498	51562517	-0.32	7.4E-38	*NKAIN1*	1	31652592	31712401	-0.18	1.1E-05	2.6E-05	-0.07	0.62
rs6958572	7:97789351	G/A	*BHLHA15*	7	97840739	97842291	0.25	2.6E-18	*PUS10*	2	61167357	61245394	-0.05	2.8E-05	2.8E-05	-0.03	0.51
rs10993994	10:51549496	C/T	*MSMB*	10	51549498	51562517	-0.32	7.4E-38	*SLC27A2*	15	50474393	50528592	0.10	4.0E-05	4.0E-05	0.03	0.72
rs11191385	10:104513049	G/T	*C10orf32-ASMT*	10	104614029	104661656	-0.15	6.5E-35	*TMEM121*	14	105992940	105996539	-0.08	3.5E-05	4.7E-05	-0.03	0.61
rs10993994	10:51549496	C/T	*MSMB*	10	51549498	51562517	-0.32	7.4E-38	*SPON2*	4	1160720	1202750	0.18	4.8E-05	4.8E-05	0.04	0.79
rs9306895	2:20878153	T/C	*GDF7*	2	20866424	20873418	-0.20	2.9E-16	*CHGB*	20	5892076	5906007	0.28	4.9E-05	4.9E-05	0.12	0.56
rs1983891	6:41536427	C/T	*FOXP4*	6	41514164	41570122	-0.09	2.0E-12	*DHX30*	3	47844399	47891685	-0.02	5.2E-05	5.2E-05	-0.01	0.50
rs1983891	6:41536427	C/T	*FOXP4*	6	41514164	41570122	-0.09	2.0E-12	*PPP2R1A*	19	52693292	52730687	-0.02	5.3E-05	5.3E-05	-0.01	0.52
rs10993994	10:51549496	C/T	*MSMB*	10	51549498	51562517	-0.32	7.4E-38	*MUC13*	3	124624289	124672663	-0.32	1.6E-05	5.9E-05	-0.13	0.61
rs58057291	2:238395479	T/A	*MLPH*	2	238394071	238463961	-0.20	2.9E-38	*PANK3*	5	167975500	168006605	0.04	7.7E-05	7.7E-05	0.01	0.72
rs11158289	14:61032390	A/G	*C14orf39*	14	60863187	60982261	-0.46	2.2E-28	*ACADVL*	17	7120444	7128592	-0.06	7.8E-05	7.8E-05	-0.03	0.56
rs11263762	17:36101926	G/A	*HNF1B*	17	36046435	36105237	0.15	4.4E-12	*SHH*	7	155592680	155604967	0.23	3.6E-05	8.1E-05	0.16	0.31
rs1983891	6:41536427	C/T	*FOXP4*	6	41514164	41570122	-0.09	2.0E-12	*ADD3*	10	111756126	111895323	0.06	8.6E-05	8.6E-05	0.03	0.48

[a]Major/Minor
